# Oxidative Stress in a Mother Consuming Alcohol during Pregnancy and in Her Newborn: A Case Report

**DOI:** 10.3390/antiox12061216

**Published:** 2023-06-04

**Authors:** Martina Derme, Maria Grazia Piccioni, Roberto Brunelli, Alba Crognale, Marika Denotti, Paola Ciolli, Debora Scomparin, Luigi Tarani, Roberto Paparella, Gianluca Terrin, Maria Di Chiara, Alessandro Mattia, Simona Nicotera, Alberto Salomone, Mauro Ceccanti, Marisa Patrizia Messina, Nunzia La Maida, Giampiero Ferraguti, Carla Petrella, Marco Fiore

**Affiliations:** 1 Department of Maternal Infantile and Urological Sciences, Sapienza University of Rome, 00185 Roma, Italy; 2 Dipartimento Della Pubblica Sicurezza, Direzione Centrale di Sanità, Centro di Ricerche e Laboratorio di Tossicologia Forense, Ministero dell’Interno, 00185 Roma, Italy; 3 Department of Chemistry, University of Turin, 10125 Turin, Italy; 4 SITAC, Società Italiana per il Trattamento Dell’alcolismo e le sue Complicanze, 00185 Rome, Italy; 5 National Centre on Addiction and Doping, Istituto Superiore di Sanità, 00161 Rome, Italy; 6 Department of Experimental Medicine, Sapienza University of Rome, 00185 Rome, Italy; 7 Institute of Biochemistry and Cell Biology (IBBC-CNR), Department of Sensory Organs, Sapienza University of Rome, 00185 Roma, Italy

**Keywords:** fetal alcohol spectrum disorder (FASD), prenatal alcohol exposure (PAE), oxidative stress, ethyl glucuronide

## Abstract

Fetal alcohol spectrum disorder (FASD) is a set of conditions resulting from prenatal alcohol exposure (PAE). FASD is estimated to affect between 2% and 5% of people in the United States and Western Europe. The exact teratogenic mechanism of alcohol on fetal development is still unclear. Ethanol (EtOH) contributes to the malfunctioning of the neurological system in children exposed in utero by decreasing glutathione peroxidase action, with an increase in the production of reactive oxygen species (ROS), which causes oxidative stress. We report a case of a mother with declared alcohol abuse and cigarette smoking during pregnancy. By analyzing the ethyl glucuronide (EtG, a metabolite of alcohol) and the nicotine/cotinine in the mother’s hair and meconium, we confirmed the alcohol and smoking abuse magnitude. We also found that the mother during pregnancy was a cocaine abuser. As a result, her newborn was diagnosed with fetal alcohol syndrome (FAS). At the time of the delivery, the mother, but not the newborn, had an elevation in oxidative stress. However, the infant, a few days later, displayed marked potentiation in oxidative stress. The clinical complexity of the events involving the infant was presented and discussed, underlining also the importance that for cases of FASD, it is crucial to have more intensive hospital monitoring and controls during the initial days.

## 1. Introduction

Prenatal alcohol exposure (PAE) is the leading preventable cause of developmental disabilities and congenital abnormalities [1,2]. Fetal alcohol spectrum disorder (FASD) is an umbrella term describing the plethora of conditions resulting from PAE [3]. FASD comprises alcohol-related neurodevelopmental disorder (ARND), alcohol-related birth defects (ARBD), fetal alcohol syndrome (FAS), and partial fetal alcohol syndrome (pFAS) [4]. FASD is thought to affect between 2% and 5% of people in Western Europe and the United States [5]. Over the years, different diagnostic guidelines for FASD have been developed; among the most recent are the guidelines by Hoyme [6], used in our case report.

Both animal models and clinical investigations have demonstrated that ethanol (EtOH) distributes through the placenta and diffuses quickly into the fetus [7]. Fetal blood alcohol levels approach maternal levels within two hours of maternal intake [8]. Once alcohol has reached the fetal circulation, the fetus attempts to metabolize EtOH through pathways similar to those of the adult [7,9]. Although the metabolic ability of the fetal liver is limited, the available findings demonstrate that the key enzyme involved in EtOH oxidation—alcohol dehydrogenases (ADH)—is present in the fetus at 2 months of gestation [9,10]. However, the metabolic capability of the fetus is severely reduced, working at a rate of about 5 to 10% of adult metabolic activity. Upon exposure, the fetus removes the EtOH through two different mechanisms: renal and pulmonary. The fetus’s reduced capability to metabolize EtOH potentiates the duration of exposure. Despite the fetus’s capability, although reduced, to remove EtOH as pulmonary and renal excretions, EtOH remains present in the amniotic fluid, leading to reabsorption, prolonging too the exposure time. Fetal swallowing has begun by 11 weeks of gestation and represents the main reabsorption component providing a path for EtOH reentry into the fetal circulation. The amniotic fluid amount swallowed by the fetus (500 to 1000 mL per day) does not account for the quantity of pulmonary secretions and urine (970 to 1370 mL per day) entering the amniotic fluid [11]. 

There are many different proposed mechanisms of alcohol teratogenicity [12]. EtOH can compromise endogenous antioxidant capacity, for example, by decreasing glutathione peroxidase levels or generating free radicals. Free radicals and reactive oxygen species (ROS), such as superoxide (O_2_^−^) and hydroxide (HO^−^) ions, are generally considered to be responsible for fetal brain damage by inducing uncontrolled apoptosis [13,14,15]. FAS facial morphology is probably linked to the apoptotic effects of alcohol on cranial neural crest cells [16]. 

The neuropsychiatric effects of FASD may be explained by EtOH, favoring the apoptosis of serotonergic neurons as shown in a mouse model [17]. FASD features appear differently in every child, but all FASD children possess behavioral and/or intellectual damage [18,19]. These children often have disrupted cognitive functioning leading to problems following directions, learning disabilities, poor memory skills, hyperactive behavior, speech and language delays, inattentiveness, and difficulties in understanding the consequences of their actions [4,20]. 

Many FASD individuals have also anomalous facial features such as a smooth ridge between the nose and upper lip, a flat nasal bridge, short palpebral fissures, extra crease in the outer ears, a thin vermillion border of the upper lip, an upturned nose, and a curved pinky finger [21]. Many children born with FASD display a smaller head size, shorter than average height, and low body weight [22]. In addition to these physical features, there are numerous medical matters that seem to be caused by fetal alcohol exposure. 

There is a strong association between alcohol use during pregnancy and congenital heart defects [23,24]. Significant associations were reported with atrial and ventricular septal defects. Furthermore, mothers abusing alcohol during gestation have a 1.64-fold times elevated risk of having an infant affected by conotruncal defect subtypes such as Great Arteries transposition. These data suggest that both binge drinking and prenatal heavy drinking are intensely associated with an overall elevated risk of having babies with congenital heart defects [25]. There is no a direct FASD cure, but early intervention and life-long support may help those born with FASD to cope with the complications that come with it [26]. 

It should be noted that recent studies refer to FASD and the fetal programming theory as a new concept [27,28]. This transformation expands FASD from being solely a neural disorder to a “whole body disorder” that affects multiple organs and systems [27,28]. This, in turn, increases the potential risk for developing chronic conditions such as cardiovascular disease (CVD) or diabetes later in life. According to these considerations, it becomes even more crucial to prioritize intensive hospital monitoring and controls during the initial days of infants affected by FASD.

In this study, we reported a case of a mother, with alcohol abuse during pregnancy, and her newborn diagnosed with FAS, presenting both increased serum ROS and oxidative stress. A significant novelty of the present case report is the disclosure, throughout 9 months of gestation, of long-lasting substance abuse (alcohol, smoking, and cocaine) by subtle hair analyses. 

## 2. Case Report

A 29-year-old Caucasian woman (gravida 1, para 0) was referred and admitted to our hospital for the management of fetal growth restriction (FGR) and oligohydramnios at 33 weeks and 6 days of gestation. The patient had iatrogenic hypothyroidism, treated with Levotiroxine 75 mcg, due to hemithyroidectomy for a benign thyroid nodule in 2016. She started Enoxaparin 4000 UI two weeks before hospitalization. 

The patient reported smoking five cigarettes per day and consuming four glasses of wine per day during all the pregnancy. She also reported using cocaine during the first two months of pregnancy. The first ultrasound at 12 weeks of gestation described a crown-rump length (CRL) corresponding to the anamnestic gestational age. The combined test in the first trimester resulted in low risk for the main aneuploidies, and a second trimester screening at 20 weeks of gestation described normal fetal anatomy. 

Upon admission to the hospital, we performed an ultrasound exam with evidence of reduced amniotic fluid (amniotic fluid index—AFI: 73 mm) and an estimated fetal weight of 1559 g, equal to the first percentile (z-score −3.303) for gestational age [29,30,31]. Fetal Doppler showed an umbilical artery pulsatility index (PI) of 1.23, equal to the 96th percentile (z-score 1.697), middle cerebral artery PI of 1.62, equal to the 16th percentile (z-score −0.995), with the cerebroplacental ratio of 1.32, equal to the 3rd percentile (z-score −2.004) for gestational age. Antenatal corticosteroids (betamethasone 12 mg intramuscular, two doses 24 h apart) were administered in order to prevent neonatal respiratory distress syndrome [32]. 

According to the Truffle Study [33], we monitored the patient every three days with an ultrasound fetal Doppler and cardiotocographic examination twice a day. We also practiced a urine screen test for amphetamine, benzodiazepines, cocaine, methadone, opiates, and cannabinoids, with all tests resulting negative. A patient hair sample (10 cm from the root), with her consent, was collected in order to evaluate the use of alcohol, smoking, and cocaine during pregnancy. In addition, a urine sample was used in order to test alcohol abuse. 

**Figure 1:**
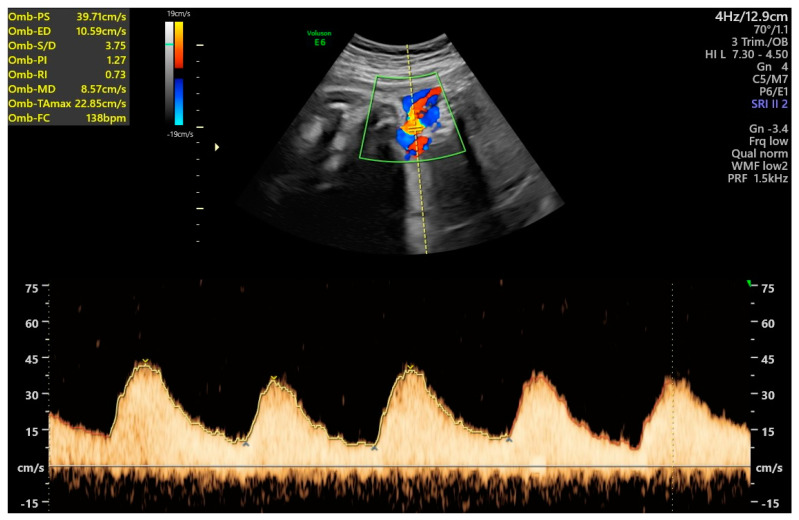
Ultrasound Doppler of the umbilical artery pulsatility index at 34 weeks and 6 days.

**Figure 2:**
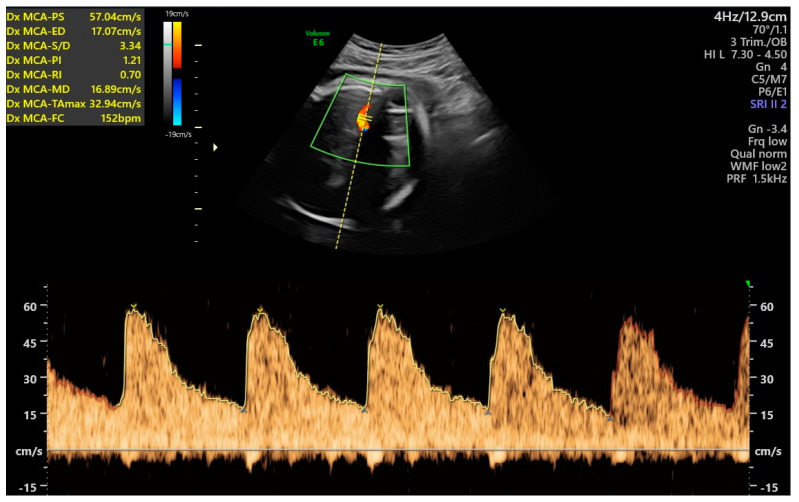
Ultrasound Doppler of the middle cerebral artery pulsatility index at 34 weeks and 6 days.

A rapid test for the diagnosis of premature rupture of membranes resulted negative. Cardiotocographic examinations always showed normal baseline heart rate, presence of accelerations, absence of decelerations, and no uterine contractions, and the short-term variability was always >4.5 ms [33]. An ultrasound exam after seven days of hospitalization (34 weeks and 6 days of gestational age) confirmed reduced amniotic fluid (AFI: 70 mm) and showed umbilical artery PI of 1.27, equal to the 98th percentile (z-score 2.005), middle cerebral artery PI of 1.21, equal to the 1st percentile (z-score −2.813), with cerebroplacental ratio of 0.95, equal to the 1st percentile (z-score −3.507) for gestational age (Figure 1 and Figure 2). 

The day after (35 weeks of gestational age), according to our local protocols, we decided for the induction of labor with vaginal Dinoprostone. Three hours after starting induction, the cardiotocography showed repetitive variable decelerations and the absence of uterine activity. The vaginal dispositive was removed after 27 min of monitoring. Intravenous fluids were administered and cardiotocography was continued. After sixty minutes of repetitive variable decelerations, loss of major accelerations, reduced baseline variability, and fetal tachycardia (mean fetal heart rate of 170 bpm), we decided to perform an emergency cesarean section. 

The day of delivery, maternal and neonatal blood samples were collected to evaluate the oxidative stress status. The newborn was a female, APGAR scores were 8 at the 1st minute and 10 at the 5th minute, pH of umbilical cord blood was 7.31, the birth weight was 1515 g (below the third percentile), the length was 39 cm (below the third percentile), and the head circumference was 29.3 cm (3° centile). The newborn was admitted to the neonatal intensive care unit (NICU) due to low birth weight (LBW), although in good general clinical condition. The urine toxicology screen came out negative.

According also to the indications of the juvenile magistrate, to avoid the risk of contamination with maternal milk, that could contain EtOH, breastfeeding was not allowed by the mother. Bottle feeding containing the standard nutritional elements of the hospital department of maternal infantile was indeed the best choice for the baby.

A physical examination performed by the neonatologists and the pediatric geneticist was significant for smooth philtrum and thin upper vermilion border, which raised suspicion for FASD along with FGR and microcephaly. FASD diagnosis was corroborated by the detection of ethyl glucuronide (EtG) in newborn meconium. Screening for further malformations was also performed. Otoacoustic emissions, abdominal ultrasound, and whole-body X-ray were unremarkable. Echocardiography displayed a foramen ovale aneurysm with a hemodynamically insignificant left-to-right shunt. Cerebral ultrasound and ophthalmic examination showed delayed gyral and sulcal development and incomplete vascularization of the peripheral retina, respectively, the latter of which resolved with the newborn’s growth. 

Complete blood count, liver and renal function tests, and electrolytes were all within the normal limits. Fifty-two days after birth, the neurological examination resulted negative. Eighty-one days after birth, a neonatal blood sample was used in order to evaluate the oxidative stress status and the result was positive, although the day of the delivery was in normal range.

## 3. Experimental Methods and Results

### 3.1. FORT (Free Oxygen Radicals Test) Investigation

By using a special kit (Callegari, Parma, Italy) for the analysis of ROS [34,35], we measured maternal and neonatal serum oxidative stress status on the day of delivery in cord blood serum [36]. FORT is a colorimetric assay based on the ability of transition metals such as iron to catalyze, in the presence of hydroperoxides (ROOH), the formation of free radicals (reaction 1–2), which are then trapped by an amine derivative, CrNH_2_ [14,35]. The amine reacts with free radicals, forming a colored, fairly long-lived radical cation, detectable at 505 nm (reaction 3). The intensity of the color correlates directly to the number of radical compounds and the hydroperoxides concentration and, consequently, to the oxidative status of the sample according to the Lambert–Beer law [14]. According to the instructions provided by the manufacturer, FORT values below 300 units (U) indicate an optimal condition of oxidative stress, for values between 300 and 330 U, a condition of latent oxidative stress, while for values superior to 330 U, a condition of oxidative stress in progress. It should be noted that there are no established reference values for women during delivery and for newborns. On the day of delivery, the maternal results described a high oxidative stress status (475 Fort U/3.61 mmol/L H_2_O_2_), while the neonatal results were normal (<160 Fort U/<1.22 mmol/L H_2_O_2_). Eighty-one days after birth, the neonatal results were positive (471 Fort U/3.58 mmol/L H_2_O_2_). 

### 3.2. Maternal Hair EtG, Nicotine/Cotinine, and Cocaine Analyses

The EtG analysis followed the protocol described by Mattia et al. [37]. Briefly, 50 mg of hair samples was decontaminated by two consecutive washes in 5 mL of methanol and 5 mL of dichloromethane, manually shredded, and incubated overnight at 60 °C in water with EtG-D5 as the internal standard. The extract was purified with solid phase extraction polymeric cartridges, air-dried, and reconstituted with acetonitrile (CAN) and N-Methyl-N-(trimethylsilyl)trifluoroacetamide (MSTFA) as a derivatizing agent for injection in an Agilent Technologies (Santa Clara, CA, USA) 7890B gas chromatograph coupled to a 7000C tandem mass selective detector operating in EI ionization mode. Hair nicotine and cotinine analyses were performed on an aliquot of 20 mg of hair samples that were decontaminated by a sonication for 15 min in 5 mL of dichloromethane, manually shredded, incubated overnight at 60 °C in NaOH 1 M with Nicotine-D4 and Cotinine-D3 as internal standard, and purified with liquid extraction in 1 mL of dichloromethane and 1 mL of 25% K_2_CO_3_. The organic phase was recovered and added with 100 μL of methanol before being concentrated under nitrogen flow to about 100 μL for GC-MS/MS analysis performed by an Agilent Technologies 7890B gas chromatograph coupled to a 7000C tandem mass selective detector operating in EI ionization mode. For the analysis of cocaine and its metabolite, benzoylecgonine, an aliquot of 40 mg of hair was decontaminated with three washes, respectively, in methanol, dichloromethane, and methanol. The last wash was analyzed to verify the absence of external cocaine contamination. The hair was dried and finely minced before being incubated overnight at 40 °C in pH6 phosphate buffer, cocaine-D3, and benzoylecgonine-D3 as internal standards. The extract was purified with anion exchange mixed-bed solid-phase extraction (SPE) cartridges. The eluate was dried under nitrogen flow and reconstituted in 50 μL of N,O-Bis(trimethylsilyl)trifluoroacetamide (BSTFA) before the analysis. The analysis was performed with an Agilent 7890B gas chromatograph coupled to a 5977B mass spectrometer detector. In our case, the EtG results were 107 pg/mg in the first half of pregnancy; 151 pg/mg in the second half of pregnancy (cut-off < 30 pg/mg). The nicotine result was 9.7 ng/mg (cut-off: 0.16 ng/mg) and the cotinine result was 0.3 ng/mg (cut-off: 0.07 ng/mg). Cocaine and benzoylecgonine analyses were positive with a value of 6.25 ng/mg (cut-off: 0.15 ng/mg) and 24 ng/mg (cut-off: 0.12 ng/mg), respectively. 

### 3.3. EtG Determination in Maternal Urine Sample

The DRI Ethyl Glucuronide Assay (W1510011723, Instrumentation Laboratory SpA, V.le Monza, 338, 20128 Milan, Italy, www.werfen.com; accessed on 20 May 2023) used in this study is intended for the qualitative and semiquantitative determination of EtG in human urine at a cut-off of 100 ng/mL as a direct value of alcohol consumption, as described by the manufacturer [38]. Briefly, this immunoenzymatic assay uses specific antibodies which can detect EtG without generating significant cross-reactivity with other glucuronic compounds. The test is based on the competition between the drug conjugate with the glucose-6-phosphate dehydrogenase (G6PDH) and the free drug in the urine sample for a fixed number of specific binding sites for the antibody [39]. In the absence of free drug in the sample, the antibody binds to the drug conjugate with G6PDH, causing a decrease in enzyme activity. This phenomenon creates a direct relationship between the concentration of the drug in urine and enzyme activity. Active enzyme converts the NAD to NADH, producing an alteration in absorbance, which can be measured with a spectrophotometric examination at 340 nm [38]. The cut-off set at 100 ng/mL was used to discriminate exposure to alcohol from non-beverage sources, or incidental exposure, which can lead to false positives [39]. The sources of possible exposure in the environment may include alcohol in mouthwash, foods, over-the-counter medications containing ethanol, and even the inhalation of alcohol from topical use [39]. In our case, the EtG in the urine sample was 124 ng/mL.

### 3.4. EtG Determination in Neonatal Meconium 

The EtG in neonatal meconium was determined according to the methods previously described [40]. Briefly, 1 mL of acetonitrile and 2 μL EtG-D5 internal standard solution were added to 100 mg of meconium and ultrasonicated at 40 °C for ten minutes. Solid-phase extraction was performed using Bond Elut-NH2 cartridges, and EtG was eluted with 1 mL HCl methanolic solution (2% *v*/*v*). The eluate was dried under nitrogen stream and derivatized with N,O-Bis(trimethylsilyl)trifluoroacetamide solution (BSTFA + 1%TMS) at 70 °C for 30 min. The extract was injected into a 7890B GC chromatographic system coupled to a 7000C triple quadruple mass spectrometer operating in multiple reaction monitoring mode acquisition [40]. EtG quantification was performed using six-point calibration curves linear in the range from 10 to 200 ng/g with a determination coefficient greater than 0.99. The bias and precision values were within the ranges acceptable in forensic toxicology (±20% bias; <20% Coefficient of Variation) [41,42]. In our case, the detection of EtG in newborn’s meconium was 37 ng/g (cut off as limit of detection, 10 ng/g).

## 4. Discussion

In this case report, we show that in a mother smoking cigarettes, drinking alcohol, and abusing cocaine during pregnancy, serum oxidative stress was strongly potentiated during delivery. We also disclosed that her newborn displayed FASD facies with in-range serum oxidative stress during delivery. However, a marked potentiation in oxidative stress measured as serum FORT was clearly evidenced in the newborn a few days after delivery. This difference is truly interesting, considering that childbirth is a physiologically stressful event in human life, and stress is usually associated with an increase in oxidative stress. The observed oxidative stress elevation in the mother during delivery could be both explained by a direct effect of the discovered gestational abuse in smoking, drinking, and using cocaine, or in combination with the stressful event of the labor and delivery. The increase in oxidative stress in the newborn disclosed a few days after birth may indicate a further long-lasting effect of the mother’s substance abuse. However, the stress induced in the newborn by hospitalization in the pediatric department after delivery for early treatments should be considered. 

It should be noted that no breastfeeding was allowed to avoid contaminations with the maternal milk that could contain EtOH. Indeed, in experimental preclinical studies, it has been described that when mothers consume ethanol during the lactation period, the effects on the offspring can be even more detrimental compared to if the mother consumed ethanol during gestation. Therefore, despite the fact that there are less studies analyzing the effects of EtOH consumption during breastfeeding and the consequent damage caused to children, it has been found in rats that the pups exposed to EtOH only during weaning presented a lower body weight at the end of breastfeeding than those who were exposed to EtOH only during gestation [43]. Furthermore, as shown in numerous human and animal model studies, malnutrition can be accompanied with alcohol and cocaine abuse during pregnancy [44,45,46,47,48]. Indeed, deficiency in nutrients in utero may also play a role in the developmental disorders related to oxidative stress and prenatal alcohol exposure. 

As for oxidative stress in FASD, oxidative stress is a condition determined by an imbalance between antioxidant and oxidative factors [13]. Oxidative stress may elicit both necrosis and cell apoptosis, determining increased carcinogenesis, cellular aging, increased autoimmune diseases, vascular stiffening, and muscle decay. In the framework of pediatric syndromes, including FASD, oxidative stress may play a subtle role in the first order [13]. Preclinical studies have indeed shown that gestational alcohol exposure disrupts the ability of specific potassium channels to dilate cerebral arterioles, which appears to be mediated also by increases in oxidative stress [49]. Furthermore, neonatal ethanol exposure induces deficits in depressive-like behavior and in context-dependent fear learning in a rat model that were associated with elevated oxidative stress findings in the prefrontal cortex and hippocampus [35].

FASD is a severe neonatal condition due to PAE [50,51,52]. Damage deriving from PAE can be permanent and have no cure, so proper identification and early treatment can help to prevent and mitigate neurological consequences that can affect the individual later in life. The severity of FASD depends on the quantity of alcohol consumed and the frequency of drinking, as well as the gestational age at which the alcohol was consumed by the mother [53,54,55,56]. Intervention services for the prevention and sensibilization of mothers may reduce the incidence of FASD. FASD is entirely preventable by avoiding alcohol. Since there is not a specific pharmacologic cure to treat alcohol-induced damage, early primary and secondary interventions are the only tools which can be used to care for children with FASD. 

Diagnosing FASD in patients requires a thorough assessment, and proof of alcohol abuse during pregnancy may help clinicians in the differential diagnosis with other syndromic conditions. In the neonatal phase, FASD may be disclosed by the presence of a small bay for the gestational age (SGA), with microcephaly and typical dysmorphic characters (short palpebral fissures, middle-facial hypoplasia, thin upper lip, telecanthus, jaw hypoplasia, elongated and flat nasolabial filter, and anomalies of the ears) [57,58,59,60]. In the neonatal or early pediatric period, heart defects can be detected. Central nervous system (CNS) anomalies, neurobehavioral conditions, and cognitive impairment represent some of the worst consequences which can be detected later in the stages of the development of the infant/adolescent. 

The exact teratogenic mechanism of alcohol on fetal development is still unclear. EtOH contributes to the malfunctioning of the neurological system in children exposed in utero by decreasing glutathione peroxidase action, with an increase in the production of ROS [30,31,61]. It causes oxidative stress and the oxidation of polyunsaturated fatty acids with the formation of aldehyde toxicants [14]. Acute and chronic alcohol exposure during prenatal development also results in impaired mitochondrial morphology and function, another important cause of cell oxidative stress. Most recent studies point to mitochondria as the potential link between prenatal alcohol exposure and brain damage [62]. 

In a mouse model, extended PAE generates an increased fraction of immature mitochondria in the fetal brain [63]. Depressed mitochondrial functioning is detected in the early postnatal phase in brain and liver tissues, including the cerebellar neurons of rats exposed prenatally. Mitochondria dysfunctions play a subtle role in the progressive decline detected during aging, in the development of neurocognitive disorders (such as Alzheimer’s disorder), and substance abuse [64]. Moreover, EtOH can directly alter the function of neurons in the CNS by disrupting the synthesis and release of neurotrophic factors, such as brain-derived neurotrophic factor (BDNF) and nerve growth factor (NGF) [65,66]. These neurotrophic factors play important roles in the growth, nutrition, survival, and development of neuronal and non-neuronal cells.

Indeed, during fetal life, disruptions in BDNF and NGF may affect the development of the limbic system, with long-lasting changes in neuronal connections and predisposition to neuropsychiatric disorders [67,68]. To the best of our knowledge, few studies have been conducted on human beings, and most of the available data are based on studies conducted on animals. The aim of our research was to provide a starting point for future studies on a larger scale in order to clarify other physiopathological mechanisms leading to FASD and to understand the real role of EtOH in oxidative stress processes and subsequent neurocognitive impairment in children with FASD. 

The main limitation of this study is the fact that there are no established FORT reference values for women during delivery and for newborns.

## 5. Conclusions

In conclusion, (i) these data do underline once again the importance, on the part of pregnant women, of carrying on the pregnancy in the healthiest way possible, avoiding any type of unnecessary stress, which includes the consumption of alcohol, smoking, and, of course, any type of drugs, as well as (ii) the importance of postnatal intensive hospital monitoring and controls in children when FASD is suspected.

## Data Availability

Data are available on request.
